# Associations between Food Outlets around Schools and BMI among Primary Students in England: A Cross-Classified Multi-Level Analysis

**DOI:** 10.1371/journal.pone.0132930

**Published:** 2015-07-17

**Authors:** Julianne Williams, Peter Scarborough, Nick Townsend, Anne Matthews, Thomas Burgoine, Lorraine Mumtaz, Mike Rayner

**Affiliations:** 1 British Heart Foundation Centre on Population Approaches for Non-Communicable Disease Prevention, Nuffield Department of Population Health, University of Oxford, Old Road Campus, Oxford, OX3 7LF United Kingdom; 2 UKCRC Centre for Diet and Activity Research (CEDAR), Medical Research Council (MRC) Epidemiology Unit, University of Cambridge School of Clinical Medicine, Institute of Metabolic Science, Cambridge Biomedical Campus, Cambridge, CB2 0QQ United Kingdom; School of Public Health, Zhejiang University, CHINA

## Abstract

**Introduction:**

Researchers and policy-makers are interested in the influence that food retailing around schools may have on child obesity risk. Most previous research comes from North America, uses data aggregated at the school-level and focuses on associations between fast food outlets and school obesity rates. This study examines associations between food retailing and BMI among a large sample of primary school students in Berkshire, England. By controlling for individual, school and home characteristics and stratifying results across the primary school years, we aimed to identify if the food environment around schools had an effect on BMI, independent of socio-economic variables.

**Methods:**

We measured the densities of fast food outlets and food stores found within schoolchildren’s home and school environments using Geographic Information Systems (GIS) and data from local councils. We linked these data to measures from the 2010/11 National Child Measurement Programme and used a cross-classified multi-level approach to examine associations between food retailing and BMI z-scores. Analyses were stratified among Reception (aged 4-5) and Year 6 (aged 10-11) students to measure associations across the primary school years.

**Results:**

Our multilevel model had three levels to account for individual (n = 16,956), home neighbourhood (n = 664) and school (n = 268) factors. After controlling for confounders, there were no significant associations between retailing near schools and student BMI, but significant positive associations between fast food outlets in home neighbourhood and BMI z-scores. Year 6 students living in areas with the highest density of fast food outlets had an average BMI z-score that was 0.12 (95% CI: 0.04, 0.20) higher than those living in areas with none.

**Discussion:**

We found little evidence to suggest that food retailing around schools influences student BMI. There is some evidence to suggest that fast food outlet densities in a child’s home neighbourhood may have an effect on BMI, particularly among girls, but more research is needed to inform effective policies targeting the effects of the retail environment on child obesity.

## Introduction

Children in England are struggling to meet healthy diet and body weight recommendations [1,2]. According to the most recent estimates, 9.3% of English children in school Reception year (age 4–5 years) and 18.9% in Year 6 (age 10–11 years) are currently obese [3]. Obesity tends to track into adulthood [4] and is difficult to reverse [5,6], which make a strong case for prevention.

A growing body of research evidence considers how obesogenic environments [7], which promote energy intake and constrain energy expenditure, may contribute to obesity risk [8–12]. While research has traditionally focused on environmental exposures near home, there has been increased interest in non-residential environments [13]. Systematic reviews indicate that associations between food retailing and diet [14] or weight status [15] vary across settings and populations and one setting of particular interest is the retail environment around schools. Policy-makers increasingly see school neighbourhoods as a logical place for health promotion as obesity prevention begins in childhood [16–19], schools are well-controlled environments and school-based interventions provide unparalleled access to children because they spend more of their waking hours in school than any other environment outside of home [20].

A systematic review of studies examining the relationship between food retailing near schools and children’s food purchases, consumption and body weight, found little evidence for an effect of retailing on purchases and consumption, but some evidence of an effect on body weight [2]. However, research in the area is still developing, meaning heterogeneous study designs, methods and measures make it difficult to draw firm conclusions about the effect that retailing near schools may have on a child’s obesity risk [21,22]. The lack of evidence about environmental effects on obesity risk is complicated further by the fact that BMI is the consequence of energy balance over time, so the effect of environmental influences may not be immediately apparent. Most of the previous studies have taken place in North America, but food environments and their effects are likely to vary between countries [23], so findings may not be generalisable. Despite these research limitations, planning authorities assume the built environment’s contribution to the obesity epidemic [24–26]. In the UK, local authorities have already implemented zoning or licensing restrictions related to hot food takeaway retailing around schools [27], supported by guidelines from the Academy of Medical Royal Colleges [28], Public Health England [29] and the Greater London Authority [30], among others.

While the focus in policy discussions [28] and research [31–34] has been on fast-food and takeaway outlets [35], it is important to consider other sources of convenient, energy-dense foods such as grocery stores, convenience stores or petrol stations. A study in New York found that the most frequent sources of food around public schools were small groceries selling mostly packaged food [36]. A recent study on energy intakes of US children aged 6 to 11 years found that 63% of energy came from stores, while only 12% came from fast food outlets [37]. Another recent study examined sources of empty calories (the sum of energy from added sugar and solid fat) across retail food stores, schools and fast-food restaurants in the US found that 33% of children’s empty calories came from stores [38]. In the UK, several pilot studies suggest that food stores on the journey to and from school [39] or near schools [40] may be a major source of calories for school children. Food outlets also tend to cluster around schools [41–43]. In England, longitudinal evidence suggests that numbers of food stores (grocers and convenience stores) and takeaway food outlets in close proximity to schools have increased in recent years [44].

In this study we investigated associations between the density of food outlets in both school and home environments and body weight in a large sample of primary school students in Berkshire, England. We examined if associations varied between types of food outlets. Additionally, we identified if associations were stronger for Reception (ages 4–5) or Year 6 (ages 10–11) students. We hypothesised that, given that Reception students have not been exposed to the school environment as long as Year 6 students and have less independence than year 6 pupils, the retail outlets around schools are unlikely to have as much of an impact as they would for year 6 students. For this reason, we hypothesised that if the Reception analysis showed null results but the year 6 analyses showed positive results, this may suggest that the food environment around schools has an influence.

## Methods

### Data

#### Individual student characteristics

We used data from the 2010/2011 National Child Measurement Programme (NCMP), which works with local authorities annually to collect data on more than one million children in state-maintained primary schools in England. The NCMP includes measures from 90% of pupils reported eligible to be measured by the primary care trusts. It includes individual height and weight (measured by health professionals, usually school nurses), sex, ethnicity and month of measurement [45]. The NCMP is an anonymized dataset, based around a no consent data collection system. Ethics approval is not required for data collection or analysis. As our primary outcome measure, we used body mass index (BMI, kg/m^2^) to calculate BMI z-scores relative to the International Obesity Task Force reference curves [46].

#### School and home characteristics

The NCMP provides each child’s school name and the lower super output area (LSOA) of the home address. LSOAs are a small geographic boundary with a mean population of 1,500 residents [47]. Using data from the Department of Education’s 2010/2011 census, we ascertained school location, size (total number of pupils), percentage of students eligible for free school meals and school type (community, foundation, voluntary-controlled or voluntary-aided) [48]. There are different types of maintained or state schools in England: Community schools are controlled by local councils and are not influenced by business or religious groups; foundation schools have more independence to change the way they do things than community schools; voluntary-controlled or voluntary-aided schools are linked to a variety of organisations including faith or charitable organisations [49]. For each child’s home LSOA, we identified the urban/rural classification [50] and child well-being index (CWI). The CWI is a composite score of domains including material well-being, health, education, crime, housing, environment and children in need (information about children who are in various kinds of need and served by local authorities, derived from the Children in Need Survey, from the Department for Children, Schools, and Families) [51].

#### Density of food outlets in school and home environments

We requested food outlet location data from the local councils of Bracknell Forest, Reading, Slough, West Berkshire, Windsor and Maidenhead, and Wokingham. Environmental health departments of local councils are required to provide this information under the Freedom of Information Act [52]. Food retailers are legally required to register their business with local councils and therefore, this has been found to be a reliable source of food outlet location information in the UK [53,54]. The local councils provided the names and addresses of all individuals, businesses and associations holding a food license.

The local council data included records of food outlets that were not of relevance to our research question (for example, industrial food manufacturers or bed and breakfasts), so we established a protocol for cleaning the data and categorizing the food outlet types. Food outlets were grouped into two categories: ‘takeaway and fast food outlets’ or ‘food stores’. We defined takeaway and fast food outlets (henceforth referred to as ‘fast food outlets’) as those selling hot or prepared food paid for before consumption [54,55], and which may be consumed off-site, a category that included coffee shops, cafes, pizza shops, sandwich shops, bakeries, delis, kebab shops and takeaway restaurants. We defined food stores as other retail outlets selling food that may be consumed off-site such as supermarkets, convenience stores, off-license stores or newsagents. Researchers consulted local business directories and Google Street View (Mountain View, California, USA) to confirm food outlet types. One researcher completed the initial processing. To test the reliability of our food outlet definitions, three researchers independently classified a 10% sample of the food outlets and there was agreement for all three raters on 88% of occasions, which gave a kappa score of 0.84.

Schools and food outlets were geocoded according to their postcode using a geographic information system (ArcGIS 10, ESRI, Redlands, CA, USA). Postcodes in the UK contain only 15 addresses on average, and therefore allow for relatively precise geocoding. Postcodes, transportation networks and LSOA boundary data were obtained from Ordnance Survey via UK Digimap (University of Edinburgh, Scotland, UK). We calculated the density of fast food outlets and food stores located within an 800 metre (m) street network buffer of school centroids and within home LSOA boundaries. Precedent for the use of an 800m buffer to represent a ‘neighbourhood’ has been set in the literature, and approximately corresponds to a 10 minute walk [2].

To test the validity of local council food outlet data, two researchers selected a random 10% sample of schools and identified the food outlets falling within an 800m street network buffer using Google Street View for comparison using percentage agreement statistics. We found that 85% (157 out of 184) of the local council-provided food outlets were also found in Google Street View. However, we also found 22 food outlets in Google Street View that were not included in the local council dataset, so when we compared agreement between the full dataset (using both sources), there was a 76% agreement.

### Statistical analysis

Using a cross-classified multi-level model, which allowed us to account for the nested structure of the data (i.e. children within school and home neighbourhoods), we examined the association between pupil BMI z-score and density of fast food and food stores in both home and school areas while controlling for confounding factors at the individual student, school and home environment levels. We categorised food outlet densities into ‘0 outlets’ (the reference category) and divided the remaining densities into tertiles. The individual-level student variables were sex, age, ethnicity and month of measurement. Home neighbourhood-level variables were urbanicity and residential child well-being index. School neighbourhood-level variables were school size, type and percentage of students on free meals.

The results for BMI are reported as z-scores in comparison to the International Obesity Task Force standard [56].

Three-level cross-classified random effects models were run using Markov chain Monte Carlo methods. Four models were calculated for each year group: Model 1 (the null model) was run for each exposure variable only (fast food near schools, food stores near schools, fast food in home neighbourhood and food stores in home neighbourhood) and controlled for no covariates, this allowed us to examine any associations between BMI z-score and food density at schools and home in univariable models. Model 2 included home and school neighbourhood covariates, and were run for each outcome variable separately, allowing us to study if any associations in Model 1 were due to confounding factors at the school or home neighbourhood level. Model 3 was also run for each outcome variable and controlled for all covariates including those at the school, home and individual levels, to investigate if any significant associations could be explained by differences between schools and home neighbourhoods in terms of pupil composition. The full model 4 included the school, home and individual level covariates, and all outcome variables together; this final model investigated whether food store densities around schools and homes had independent associations with BMI z-score. Ninety five percent confidence intervals (CIs) were used to determine significant differences between tertiles of outlet density and BMI z-score. We also used Wald tests to determine whether the non-reference categories were significantly different to each other in their association with the outcome variable. All analyses were conducted using MLwiN version 2.28 (Centre for Multilevel Modelling, University of Bristol, UK).

## Results

### Descriptive Statistics

We analysed anonymised data from 16,956 individual children attending 268 schools, and living in 664 different home neighbourhoods in Berkshire. Descriptive statistics for the study sample are shown in Table 1. The mean BMI z-score for Reception students (n = 8,745) was 0.38 (*SD* = 1.00) and for Year 6 students (n = 8,211) was 0.51 (*SD* = 1.04); these are both lower than the mean BMI z-score found for the national sample in the same calendar year (Reception = 0.46, Year 6 = 0.56). Descriptive statistics for home and school neighbourhood factors, as well as BMI z-scores by home and school neighbourhood characteristics are shown in Table 2. The majority of home neighbourhoods (80.3%) were classified as ‘urban city and town’, and more than half (57.1%) of the participating schools were community schools. Pupils attending schools with a greater proportion of students eligible for free school meals had higher mean BMI z-scores.

**Table 1:** Frequencies and mean BMI z-scores for individual-level factors.

**Descriptive statistics**	**Reception**	**Year 6**	**Total**	**BMI z-score**	**Reception**	**Year 6**
					**Mean (SD)**	**Mean (SD)**
**Individual- n**	8,745	8,211	16,956	All	0.38 (1.00)	0.51 (1.04)
**Sex—(%)**				**Sex**		
Male	4,501 (51.5)	4,265 (51.9)	8,766 (51.7)	Male	0.34 (1.00)	0.49 (1.05)
Female	4,244 (48.5)	3,946 (48.1)	8,190 (48.3)	Female	0.42 (1.00)	0.52 (1.04)
**Age in months**				**Age quartiles**		
Mean (SD)	63.0 (5.3)	133.4 (3.8)	97.1 (35.5)	1st Quartile	0.43 (0.98)	0.51 (1.04)
Min-Max	48.9–77.6	124.5–142.3	48.9–142.3	2nd Quartile	0.42 (0.98)	0.49 (1.03)
				3rd Quartile	0.34 (0.99)	0.52 (1.06)
				4th Quartile	0.32 (1.03)	0.51 (1.04)
**Ethnicity—(%)**				**Ethnicity**		
Bangladeshi	14 (0.2)	42 (0.5)	56 (0.3)	Bangladeshi	0.02 (1.16)	1.01 (1.24)
Black African	90 (1.0)	228 (2.8)	318 (1.9)	Black African	0.39 (1.19)	0.76 (1.04)
Black Caribbean	25 (0.3)	75 (0.9)	100 (0.6)	Black Caribbean	-0.26 (0.84)	0.66 (1.24)
Chinese	10 (0.1)	49 (0.6)	59 (0.3)	Chinese	0.21 (0.55)	0.22 (1.12)
Indian	195 (2.2)	410 (5.0)	605 (3.6)	Indian	0.10 (1.16)	0.58 (1.15)
Pakistani	363 (4.2)	591 (7.2)	954 (5.6)	Pakistani	0.08 (1.16)	0.57 (1.27)
White British	1,789 (20.5)	5,520 (67.2)	7,309 (43.1)	White British	0.39 (0.89)	0.46 (0.99)
White Irish	19 (0.2)	44 (0.5)	63 (0.4)	White Irish	0.58 (0.94)	0.67 (0.88)
Other	398 (4.6)	1,169 (14.2)	1,567 (9.2)	Other	0.35 (1.11)	0.62 (1.07)
Not stated	5,842 (66.8)	83 (1.0)	5,925 (34.9)	Not stated	0.41 (0.99)	0.39 (1.09)
**Month measured—n (%)**				**Month measured**		
Sept—Dec	2,532 (29.0)	0 (0)	2,532 (14.9)	Sept—Dec	0.40 (1.02)	-
Jan—April	4,313 (49.3)	4,851 (59.1)	9,164 (54.0)	Jan—April	0.38 (0.98)	0.49 (1.03)
May—Aug	1,900 (21.7)	3,360 (40.9)	5,260 (31.0)	May—Aug	0.34 (0.99)	0.54 (1.06)

**Table 2:** Frequencies and mean BMI z-scores for home- and school-level factors.

**Home Lower Super Output Area (LSOA) Factors**						
**Descriptive Statistics**	**Reception**	**Year 6**	**Total**	**BMI z-score**	**Reception**	**Year 6**
LSOA—n	614	628	664		Mean (SD)	Mean (SD)
**Urban/rural—(%)**				**Urban/rural**		
Rural town and fringe	52 (8.5)	52 (8.3)	57 (8.6)	Rural town and fringe	0.42 (0.33)	0.54 (0.56)
Rural village and dispersed	43 (7.0)	45 (7.2)	51 (7.7)	Rural village and dispersed	0.37 (0.36)	0.22 (0.57)
Urban city and town	509 (82.9)	516 (82.2)	533 (80.3)	Urban city and town	0.36 (0.33)	0.53 (0.41)
Urban major conurbation	10 (1.6)	15 (2.4)	23 (3.5)	Urban major conurbation	0.72 (0.89)	0.64 (1.03)
**Child Well Being Index**				**Child Well Being Index**		
Mean (SD)	100.4 (73.5)	99.5 (73.3)	99.5 (72.79)	1st Quartile	0.33 (0.36)	0.45 (0.48)
Min-Max	9.2–400.8	9.2–400.8	9.2–400.8	2nd Quartile	0.30 (0.33)	0.46 (0.47)
				3rd Quartile	0.44 (0.36)	0.50 (0.49)
				4th Quartile	0.43 (0.32)	0.61 (0.47)
**School-level factors**						
**Descriptive Statistics**	**Reception**	**Year 6**	**Total**	**BMI z-score**	**Reception**	**Year 6**
**School-n**	238	221	268		**Mean (SD)**	**Mean (SD)**
**School type—(%)**				School type		
Community	135 (56.7)	125 (56.6)	153 (57.1)	Community	0.38 (0.21)	0.51 (0.22)
Foundation	6 (2.5)	6 (2.7)	6 (2.2)	Foundation	0.38 (0.32)	0.72 (0.28)
Voluntary aided	55 (23.1)	54 (24.4)	62 (23.1)	Voluntary aided	0.36 (0.23)	0.47 (0.28)
Voluntary controlled	42 (17.6)	36 (16.3)	47 (17.5)	Voluntary controlled	0.41 (0.23)	0.50 (0.31)
**Total no. Pupils—(%)**				**Total no. Pupils**		
Mean (SD)	268.9 (150.0)	285.4 (150.6)	276.8 (144.3)	1st Quartile	0.38 (0.27)	0.44 (0.31)
Min-Max	29.0–763.0	29.0–763.0	29.0–763.0	2nd Quartile	0.38 (0.22)	0.49 (0.24)
				3rd Quartile	0.41 (0.21)	0.56 (0.20)
				4th Quartile	0.37 (0.21)	0.53 (0.23)
**Free School Meals—n (%)**				**Free School Meals**		
Mean (SD)	10.1 (8.8)	10.5 (9.4)	10.1 (9.0)	1st Quartile	0.30 (0.20)	0.37 (0.21)
Min-Max	0.0–46.7	0.0–48.2	0.0–48.2	2nd Quartile	0.40 (0.21)	0.47 (0.22)
				3rd Quartile	0.41 (0.24)	0.57 (0.26)
				4th Quartile	0.42 (0.23)	0.61 (0.24)

Food outlet frequencies within home and school neighbourhoods are shown in Table 3. The number of fast food outlets found within home neighbourhoods ranged from 0 to 35, with a mean of 1.14 (*SD* = 3.05). The number of food stores ranged from 0 to 35, with a mean of 1.40 (*SD* = 3.03). There were more fast food outlets and food stores in the most deprived home neighbourhoods (i.e. those falling within the highest quartiles of the Child Well Being Index). The number of fast food outlets found within an 800 metre street network of schools ranged from 0 to 30 outlets, with a mean of 2.67 (*SD* = 4.47). The number of food stores ranged from 0 to 33, with a mean of 3.34 (*SD* = 4.57). There were more fast food outlets and food stores located near larger schools and those with the highest proportion of students eligible for free school meals (Table 3).

**Table 3:** Food outlet frequencies by home and school neighbourhoods.

**Food outlets within home Lower Super Output Area (LSOA)**		
Store Frequency	Fast food (n = 630)	Food stores (n = 630)
	*Mean (SD)*	*Mean (SD)*
	1.14 (3.05)	1.40 (3.03)
**Child Well Being Index**		
1st Quartile	0.74 (2.95)	1.05 (2.74)
2nd Quartile	0.88 (2.17)	1.14 (2.20)
3rd Quartile	1.50 (4.03)	1.72 (2.67)
4th Quartile	1.49 (2.72)	1.72 (2.66)
**Urban/rural**		
Rural town and fringe	0.70 (1.60)	1.09 (1.71)
Rural village and dispersed	0.57 (1.41)	0.88 (1.61)
Urban city and town	1.27 (3.30)	1.52 (3.26)
Urban major conurbation	0 (0)	0 (0)
**Food outlets near schools (within 800 metres)**		
Store Frequency	Fast food (n = 712)	Food stores (n = 892)
	*Mean (SD)*	*Mean (SD)*
	2.67 (4.47)	3.34 (4.57)
**School type**		
Community	2.8 (4.4)	3.5 (4.8)
Foundation	3.8 (3.4)	5.7 (5.1)
Voluntary aided	2.8 (5.2)	3.4 (4.8)
Voluntary controlled	1.8 (3.6)	2.5 (3.1)
**Total no. Pupils**		
1st Quartile	1.0 (2.9)	1.7 (3.4)
2nd Quartile	2.0 (3.0)	2.8 (3.2)
3rd Quartile	4.3 (6.0)	4.8 (6.2)
4th Quartile	3.5 (2.7)	4.1 (4.2)
**Free Meals**		
1st Quartile	1.5 (3.6)	2.0 (3.8)
2nd Quartile	1.6 (2.6)	2.2 (3.0)
3rd Quartile	3.2 (4.9)	3.9 (4.3)
4th Quartile	4.3 (5.6)	5.2 (5.9)

A description of participant BMI z-scores by home and school neighbourhoods is shown in Table 4. Reception students living in LSOAs with no fast food outlets had a mean BMI z-score of 0.36 (*SD = 0*.*33)*, while those living in LSOAs with 30–35 fast food outlets had a BMI z score of *0*.*90 (SD = 0*.*63)*. Among Year 6 students, there was relatively less of a difference between pupils living in areas with no food outlets compared to those living in areas with the highest density of outlets, with BMI z scores of 0.51 (SD = 0.45) and 0.45 (SD = 0.43), respectively. Reception and Year 6 students attending schools with no fast food or no food stores within 800 metres had lower mean BMI z-scores than those attending schools with the highest densities of food outlets.

**Table 4:** BMI z-scores by home and school neighbourhoods.

**Food outlets within home Lower Super Output Areas (LSOAs)**									
			**BMI z-score**					**BMI z-score**	
			**Reception**	**Year 6**				**Reception**	**Year 6**
	**N-LSOAs**	%	*Mean (SD)*	*Mean (SD)*		**N-LSOAs**	%	*Mean (SD)*	*Mean (SD)*
**Fast food**			**0.37 (0.33)**	**0.51 (0.45)**	**Food stores**			**0.37 (0.34)**	**0.51 (0.45)**
0	404	64.1	0.36 (0.37)	0.49 (0.50)	0	320	50.8	0.37 (0.39)	0.51 (0.54)
1	88	14	0.37 (0.28)	0.47 (0.340	1	131	20.8	0.36 (0.28)	0.48 (0.36)
2	52	8.3	0.44 (0.29)	0.61 (0.32)	2	75	11.9	0.39 (0.28)	0.56 (0.34)
3	32	5.1	0.38 (0.28)	0.55 (0.42)	3	37	5.9	0.34 (0.30)	0.48 (0.35)
4	16	2.5	0.32 (0.30)	0.63 (0.37)	4	26	4.1	0.39 (0.30)	0.60 (0.36)
5	14	2.2	0.33 (0.18)	0.62 (0.38)	5	16	2.5	0.30 (0.29)	0.60 (0.50)
6–9	15	2.4	0.54 (0.20)	0.69 (0.31)	6–9	16	2.5	0.45 (0.28)	0.53 (0.35)
10–19	6	1	0.43 (0.22)	0.49 (0.19)	10–19	4	0.6	0.45 (0.28)	0.38 (0.16)
20–29	0	0	-	-	20–29	3	0.5	0.36 (0.22)	0.52 (0.7)
30–35	2	0.3	0.90 (0.63)	0.45 (0.43)	30–35	2	0.3	0.42 (0.07)	0.39 (0.60)
**Food outlets near schools (within 800 metres)**									
			**BMI z-score**					**BMI z-score**	
			**Reception**	**Year 6**				**Reception**	**Year 6**
	**N-schools**	%	*Mean (SD)*	*Mean (SD)*		**N-schools**	%	*Mean (SD)*	*Mean (SD)*
**Fast food**			**0.38 (0.23)**	**0.51 (0.25)**	**Food stores**			**0.38 (0.23)**	**0.51 (0.25)**
0	119	44.6	0.38 (0.24)	0.45 (0.25)	0	74	27.7	0.35 (0.23)	0.40 (0.20)
1	34	12.7	0.36 (0.24)	0.52 (0.29)	1	46	17.2	0.39 (0.22)	0.52 (0.30)
2	35	13.1	0.36 (0.18)	0.56 (0.20)	2	40	15	0.42 (0.26)	0.54 (0.24)
3	20	7.5	0.32 (0.21)	0.50 (0.25)	3	25	9.4	0.39 (0.19)	0.50 (0.18)
4	12	4.5	0.45 (0.27)	0.59 (0.19)	4	21	7.9	0.45 (0.20)	0.65 (0.27)
5	5	1.9	0.33 (0.18)	0.62 (0.08)	5	10	3.7	0.26 (0.14)	0.46 (0.25)
6–9	19	7.1	0.42 (0.15)	0.47 (0.22)	6–9	26	9.7	0.42 (0.22)	0.62 (0.23)
10–19	19	7.1	0.50 (0.24)	0.73 (0.23)	10–19	22	8.2	0.37 (0.26)	0.55 (0.24)
20–29	3	1.1	0.41 (0.09)	0.68 (0.27)	20–29	2	0.7	0.47 (0.05)	0.57 (-)
30			-	0.19 (-)	30–35	1	0.4	0.38 (-)	0.98 (-)

### Associations between the density of food outlets and BMI z-scores

#### School neighbourhoods

Results from the cross-classified multilevel analysis are shown in Tables 5 and 6. For Reception students overall, there were no significant associations between school fast food or food store densities and BMI (Table 5). For Year 6 students, initial models showed a significant positive relationship between school fast food and food store densities and BMI z-score, however these associations were null when adjusting for school, home or individual-level covariates (Table 6).

**Table 5:** Associations between food outlets in home Lower Super Output Area (LSOA) and within 800 metres of schools and BMI z-score among Reception students in Berkshire, England.

Food	Reception Students Overall								Boys		Girls	
Outlets^a^	Model 1		Model 2		Model 3		Model 4		Model 4		Model 4	
*ref = 0*	β	95% CI	β	95% CI	β	95% CI	β	95% CI	β	95% CI	β	**95% CI**
	Fast food around schools (ref = 0)											
1	-0.011	-0.08, 0.058	-0.01	-0.08, 0.061	0.005	-0.064, 0.073	-0.01	-0.086, 0.065	0.004	-0.094, 0.103	-0.028	-0.127, 0.071
2	0.019	-0.068, 0.107	0.025	-0.063, 0.113	0.041	-0.046, 0.128	0.046	-0.056, 0.147	0.055	-0.071, 0.182	0.037	-0.088, 0.163
3	0.059	-0.015, 0.133	0.048	-0.032, 0.128	0.058	-0.019, 0.135	0.113	0,0.226	0.121	-0.020, 0.263	0.087	-0.056, 0.231
Wald	2.949	(p = 0.229)	1.957	(p = 0.376)	1.854	(p = 0.396)	5.405	(p = 0.067)	2.979	0.226	2.974	0.226
	Food stores around schools (ref = 0)											
1	0.04	-0.034, 0.114	0.038	-0.039, 0.115	0.043	-0.031, 0.118	0.045	-0.034, 0.125	0.025	-0.077, 0.128	0.064	-0.037, 0.166
2	0.077	-0.008, 0.162	0.058	-0.028, 0.144	0.055	-0.03, 0.141	0.032	-0.07, 0.135	0.038	-0.093, 0.168	0.034	-0.098, 0.167
3	0.022	-0.056, 0.1	-0.006	-0.094, 0.083	0.029	-0.056, 0.113	-0.05	-0.173, 0.074	-0.069	-0.223, 0.086	-0.020	-0.179, 0.139
Wald	3.392	(p = 0.335	2.333	(p = 0.311)	0.411	(p = 0.814)	3.453	(p = 0.178)	3.056	0.217	1.538	0.464
	Fast food in home Lower Super Output Area (ref = 0)											
1	-0.012	-0.075, 0.051	-0.011	-0.073, 0.051	-0.006	-0.068, 0.056	0.015	-0.05, 0.08	0.005	-0.087, 0.097	0.022	-0.073, 0.117
2	0.067	-0.011, 0.145	0.06	-0.016, 0.135	0.058	-0.019, 0.134	**0.084** *	**0.001, 0.167**	0.043	-0.074, 0.161	**0.125** *	**0.006, 0.244** *
3	0.008	-0.051, 0.067	-0.005	-0.065, 0.055	-0.001	-0.058, 0.056	0.042	-0.031, 0.115	0.045	-0.062, 0.152	0.047	-0.057, 0.151
Wald	3.367	(p = 0.339)	2.777	(p = 0.249)	2.289	(p = 0.318)	2.286	(p = 0.319)	0.568	0.753	2.530	0.282
	Food stores in home Lower Super Output Area (ref = 0)											
1	-0.02	-0.079, 0.038	-0.03	-0.088, 0.028	-0.027	-0.085, 0.031	-0.045	-0.105, 0.014	**-0.092**	**-0.176, -0.007** *	-0.002	-0.087, 0.083
2	-0.006	-0.073, 0.062	-0.008	-0.073, 0.058	-0.006	-0.073, 0.06	-0.037	-0.11, 0.036	-0.012	-0.112, 0.089	-0.057	-0.161, 0.048
3	-0.034	-0.094, 0.025	-0.055	-0.115, 0.006	-0.048	-0.106, 0.01	**-0.086** *	**-0.162,-0.011**	-0.096	-0.202, 0.011	-0.082	-0.189, 0.026
Wald	0.588	(p = 0.745)	1.601	(p = 0.449)	1.280	(p = 0.527)	1.689	(p = 0.43)	3.034	0.219	2.384	0.304

**Model 1** Univariable models for each exposure variable, **Model 2** Univariable exposure variables with controls for school (number of pupils, percentage eligible for free meals, school type, school urbanicity) and LSOA (urbanicity and IMD), **Model 3** Multivariable- Univariable exposure with all controls (school, LSOA and pupil: sex age, month of measurement, ethnicity), **Model 4** Multivariable exposure with all controls.

^a^Food outlet densities were categorised into zero outlets (the reference group) and the remaining densities were divided into tertiles.

*Indicates significant associations (p <0.05)

**Table 6:** Associations between food outlets in home Lower Super Output Area (LSOA) and within 800 metres of schools and BMI z-score among Year 6 students in Berkshire, England.

Food	Year 6 Students Overall								Boys		Girls	
Outlets^a^	Model 1		Model 2		Model 3		Model 4		Model 4		Model 4	
*ref = 0*	β	95% CI	β	95% CI	β	95% CI	β	95% CI	β	95% CI	β	95% CI
	Fast food around schools (ref = 0)											
1	0.064	-0.012, 0.14	0.016	-0.058, 0.09	0.029	-0.05, 0.108	-0.008	-0.089, 0.073	0.002	-0.096, 0.100	-0.001	-0.110, 0.108
2	0.059	-0.039, 0.157	0.009	-0.083, 0.101	-0.002	-0.096, 0.092	-0.033	-0.142, 0.077	-0.002	-0.133, 0.129	-0.019	-0.165, 0.126
3	**0.121** *	**0.037, 0.205**	0.039	-0.041, 0.119	0.028	-0.059, 0.116	0.038	-0.094, 0.17	0.101	-0.052, 0.254	0.018	-0.152, 0.187
Wald	1.780	(p = 0.411)	0.440	(p = 0.803)	0.485	(p = 0.785)	1.546	(p = 0.462)	2.511	p = -0.285	0.241	p = -0.886
	Food stores around schools (ref = 0)											
1	0.121*	0.041, 0.201	0.059	-0.019, 0.137	0.073	-0.01, 0.155	0.085	-0.003, 0.174	0.034	-0.070, 0.138	0.113	0.000, 0.227
2	0.148*	0.052, 0.244	0.064	-0.032, 0.16	0.075	-0.021, 0.172	0.096	-0.017, 0.21	0.056	-0.077, 0.189	0.106	-0.043, 0.254
3	0.161*	0.077, 0.245	0.037	-0.045, 0.119	0.036	-0.051, 0.123	0.018	-0.124, 0.159	-0.053	-0.212, 0.106	0.070	-0.110, 0.250
Wald	1.109*	(p = 0.574)	0.486	(p = 0.784)	1.128	(p = 0.569)	2.288	(p = 0.319)	2.744	p = -0.254	0.297	p = -0.862
	Fast food in home Lower Super Output Area (ref = 0)											
1	0.009	-0.062, 0.08	0.013	-0.054, 0.08	0.013	-0.057, 0.084	0.024	-0.044, 0.092	-0.029	-0.127, 0.069	0.058	-0.044, 0.160
2	**0.106** *	**0.018, 0.194**	0.089	0.003, 0.175	0.079	-0.008, 0.166	**0.096** *	**0.011, 0.181**	-0.046	-0.171, 0.079	**0.246** *	**0.115, 0.377**
3	**0.117** *	**0.052, 0.182**	0.086	0.017, 0.155	**0.083** *	**0.018, 0.147**	**0.120** *	**0.042, 0.198**	0.041	-0.071, 0.153	**0.185** *	**0.067, 0.303**
Wald	**6.879** *	**(p = 0.032)**	3.519	(p = 0.172)	3.072	(p = 0.215)	4.448	(p = 0.108)	2.024	p = -0.363	**6.119** *	**p = -0.047**
	Food stores in home Lower Super Output Area (ref = 0)											
1	-0.014	-0.083, 0.055	-0.028	-0.091, 0.035	-0.028	-0.088, 0.032	-0.046	-0.108, 0.017	-0.025	-0.111, 0.061	-0.085	-0.174, 0.005
2	**0.087** *	**0.005, 0.169**	**0.084** *	**0.01, 0.158** *	**0.079** *	**0.008, 0.151**	0.032	-0.05, 0.114	0.107	-0.007, 0.221	-0.073	-0.191, 0.044
3	0.049	-0.02, 0.118	0.012	-0.053, 0.077	0.004	-0.062, 0.069	-0.073	-0.154, 0.008	0.013	-0.101, 0.127	-0.165	-0.283,-0.046
Wald	5.888	(p = 0.527)	**7.731** *	**(p = 0.021)**	**6.517** *	**(p = 0.038)**	5.331	(p = 0.070)	5.361	p = -0.069	2.238	p = -0.327

**Model 1** Univariable models for each exposure variable, **Model 2** Univariable exposure variables with controls for school (number of pupils, percentage eligible for free meals, school type, school urbanicity) and LSOA (urbanicity and IMD), **Model 3** Multivariable- Univariable exposure with all controls (school, LSOA and pupil: sex age, month of measurement, ethnicity), **Model 4** Multivariable exposure with all controls.

^a^Food outlet densities were categorised into zero outlets (the reference group) and the remaining densities were divided into tertiles.

*Indicates significant associations (p <0.05)

#### Home neighbourhoods

In model 4, Reception students living in home neighbourhoods with the highest densities of fast food had a mean BMI z-score 0.08 (95% CI: 0.00, 0.17) higher than those living in home neighbourhoods with no fast food stores. When we stratified the analyses by sex, we found that this relationship was significant for Reception girls (mean BMI z-score = 0.13, 95% CI 0.00, 0.24), but not boys. However, evidence of a dose-response effect in this relationship was not observed. Reception students whose home neighbourhoods had the highest density of food stores had a mean BMI z-score that was 0.09 (95% CI: -0.16, -0.01) lower than students living in home neighbourhoods with no food stores, however associations with other densities of home neighbourhood food store exposure were null. When we stratified the analyses by sex, we found that the negative relationship between food store densities and BMI was significant for boys but not girls (mean BMI z-score = -0.09, 95% CI: -0.18, -0.01).

There was a positive relationship between BMI z-scores and home fast food outlet density for Year 6 students. Those who were exposed to the highest densities of fast food outlets in the home neighbourhood had BMI z-scores 0.10 (95% CI: 0.01, 0.18) and 0.12 (95% CI: 0.04, 0.20) greater than those least exposed. When we stratified the analyses by sex, we found that the relationship between fast food outlets near homes and BMI z-score was significant for girls, but not for boys. Year 6 girls living areas with the highest densities of fast food had a mean BMI z-score that was 0.19 (95% CI: 0.07, 0.3) greater than those living in areas with none. Adjusted associations between BMI z-scores and school neighbourhood fast food or food store densities were null for Year 6 students.

## Discussion

This analysis did not support an independent effect of food stores or fast food outlets around schools on body weight in a sample of UK Reception or Year 6 students. However, there was evidence of a weak positive association between fast food outlet exposure around the home and body weight for older girls in this sample, which remained after adjustment for individual-level and school-level covariates, and exposure to other types of food stores.

There are a number of strengths and limitations to this paper. One of the main strengths was our use of school, home and individual-level measures, which enabled us to identify multi-level determinants of body weight while accounting for the cross-classified structure of the data [57]. The importance of accounting for individual characteristics and exposures from multiple locations is highlighted by Kestens *et al*, who found that estimates of food environment exposures accounting for both residential and non-residential settings were significantly and more strongly associated with overweight than estimates based on one exposure setting only [58]. This relationship differed between men and women, highlighting the importance of multiple estimates of environmental exposure based around key daily anchor points such as homes and schools, as well as the potential moderating influence of individual-level characteristics such as gender.

Few studies have reported on the reliability or validity of their methods to characterize food environments. We conducted inter-rater reliability tests for our food outlet inclusion criteria and categorization, finding 88% agreement, which gave a kappa score of 0.84. We also validated the data provided from the local councils against Google Street View, a virtual street audit method that has been identified as a potentially promising alternative to ground-truthing [59]. We found a low level of agreement between the two sources, which may have been due to temporal mismatch between local council records and Google’s images. Earlier attempts to validate local council food outlet data with Google Street View found similarly low levels of agreement [60].

The acceptability and validity of secondary food outlet data sources, including data from local councils in the United Kingdom, has been tested previously against field observations and has been found to be the most accurate publicly available source of food environment data compared to the ‘gold standard’ of field testing[52]. Lake et al compared three sources of food environment data (Yellow Pages, Yell.com and Council Data) to a ‘gold standard’ of observations made in the field and found that the council data had a positive predictive value of 91.5%[54]. Similarly, Cummins et al [53] found reasonable, but imperfect agreement between a publicly available directory of food retail premises and field-validated reality. However, while council data is likely to be in the upper bounds of validity compared to commercial listings (because it is collected for regulatory purposes), researchers should acknowledge the imperfect nature of such data [53].

Our study was limited by its cross-sectional design, which precluded us from establishing a causal relationship between food outlet exposure and body weight. While the NCMP data provided BMI measures for a large number of children, it did not include other data on physical activity or dietary behaviours, which may be more strongly related to the exposures examined here. We characterized food retailing in home and school neighbourhoods, but we did not capture exposures along the journey between home and school, or in wider activity space environments, which may be important [61–63]. We used 800m buffers around schools and home LSOA boundaries as a proxy for neighbourhoods, but this may not be an accurate reflection of a child’s neighborhood [63].

Questions remain about defining and classifying food outlets. In this study, we drew upon studies by Lake et al[54] and Burgoine et al [55] in forming our definitions of fast food outlets and food stores. However, definitions vary widely between studies. A review on fast food access by Fleischacker et al [64] found that close to half of the studies (n = 16, 40%) used a proprietary fast food outlet definition. Given this variation, it is important for future research to be clear about their criteria for classifying food outlets.

Food access includes many components [14] which were beyond the scope of this study to measure. Our measures of food availability (density of fast food outlets and food stores) may not reflect the foods actually available within stores. Categorizing food outlets as ‘healthy’ (e.g. grocery stores) and ‘unhealthy’ (e.g. takeaway shops) is problematic [65] because both healthy and unhealthy food options are often found within the same venue. Future research needs to integrate detailed information on what is sold within stores, alongside measures of access to the stores themselves [22]. However, it is possible that use of food stores varies as a function of age such that young children make less healthy choices than older children and adults. This hypothesis is yet to be explored in the published literature.

This study did not consider the food environments within schools, which have broad potential to impact pupil’s food choices [66–68]. School meal standards in the United Kingdom have been described as amongst some of the most detailed and comprehensive in the world[69] and recent reforms from the Department of Education have introduced a new set of standards for all food served in schools [70]. In contexts where the within-school food environment is less regulated (or where standards are non-existent), future research should consider both the within-school and out-of-school availability of food when considering environmental influences on diet quality.

The NCMP data allowed us to control for individual-level characteristics such as sex, age, and ethnicity, but it did not include other potentially important factors, such as cognitions, psychological and psychosocial factors [71]. Our school data set would have been strengthened by additional measures on socio-cultural and political environments [72].

Previous research studies on associations between food outlets around schools and obesity or diet outcomes among children have varied widely in their exposure and outcome measures [2], and have found mixed results. To the best of our knowledge, of those studies examining neighbourhood environment effects on body weight, only two have been conducted in England [73,74]. Like our study, Harrison *et al* considered food retail exposures in both school and home environments and found retailing around homes was more strongly associated with Fat Mass Index (FMI) than retailing around schools [63]. Among girls, access to unhealthy food outlets (takeaways and convenience stores) near home was associated with FMI and access to healthy outlets (supermarkets and greengrocers) near home was associated with a lower FMI. Similarly, we found high access to fast food in the home neighbourhoods of Year 6 students was associated with a higher BMI z-score and high access to food stores in the home of Reception students was associated with a lower BMI z-score. Harrison *et al* also measured the child’s mode of travel to school and found associations varied between active- and non-active travellers, with some evidence that environmental exposures may have more of an influence on the former. This has important implications for our study because children who are driven to and from school may have little chance to interact with the school neighbourhood.

Our study builds upon earlier work examining associations between food environments, deprivation and childhood overweight and obesity using NCMP data [73]. Unlike our study, which was limited to one region in England, this study included schools across England and had data from three time points (2007–2010). Our main contribution to this work is the inclusion of individual-level measures (rather than aggregate school-level measures). Also, while their study focused on takeaways, we considered takeaways and other types of retailers. Cetateanu *et al* observed associations between takeaways around schools and obesity rates among older (Year 6) students, but not the younger (Reception) students. Similarly, we found stronger evidence of associations between food retailing and BMI among older students (Year 6) compared to younger students (Reception). One possible explanation is that older children have increased autonomy, spending power and capacity to travel independently to and from school. However, Sanchez *et al* [75] found that the presence of convenience stores near schools was significantly associated with the overweight prevalence ratio of fifth grade students (around age 10), but not ninth grade students (about 14 years). The reasons behind this discrepancy are currently unclear.

We do not know why associations between fast food outlets near homes and mean BMI z scores were stronger for girls than they were for boys. Previous research has also shown sex differences in associations between the environment and weight-related outcomes [74,76–78], but further work is needed to understand what lies behind these differences.

There are many reasons why we may not have seen an association between food retailing around schools and BMI. Perhaps an association would have been observed if we had looked at more proximal outcomes like diet or physical activity. It could be that students near schools with a high density of outlets eat more fast food, but compensate for it with higher levels of physical activity or eat less food at home. Another possibility is that the cumulative effects have not yet appeared among primary school children. Unfortunately, the NCMP doesn’t measure secondary school pupils, who may be more affected by food outlets around schools than younger students. Future research on associations between food retailing and weight among secondary schoolchildren would benefit from considering whether or not schools have stay-on-site lunch policies. While this is the norm among primary schools, many secondary schools allow older students to leave campus for meals.

Future work may benefit from the collection and analysis of data over a wide range of spatial and temporal scales [13,79,80] while accounting for individual-level characteristics. Research in this area will also would benefit from using a national sample at various time points, as done by Cetateanu *et al* [73], while also controlling for individual-level and home-environment characteristics.

Additional work is needed to find ways to more accurately characterize environmental exposures at home and school [81]. Accounting for individual mobility patterns such as activity spaces [82] or GPS routes [83] will enable researchers to assess the various environments beyond home and school. One recent example in the UK found that exposure to takeaway food outlets in home, work, and commuting environments combined was associated with higher consumption of takeaway food, greater body mass index, and greater odds of obesity among adults [55].

Obesity is the consequence of a complex web of influences; its underlying systems that are non-linear and ill-suited to dichotomous hypothesis testing [84]. The scientific evidence about the effects of food outlets on child BMI is not conclusive. However, in the face of this uncertainty, proponents of the precautionary principal would argue that if plausible risk [16] to health has been identified, preventive measures are warranted.

## Supporting Information

S1 Table Food Outlets in West Berkshire: Names, Postcodes, Categories and Addresses from Local Councils. (XLSX)
