# Correction: Associations between Food Outlets around Schools and BMI among Primary Students in England: A Cross-Classified Multi-Level Analysis

**DOI:** 10.1371/journal.pone.0147164

**Published:** 2016-01-11

**Authors:** Julianne Williams, Peter Scarborough, Nick Townsend, Anne Matthews, Thomas Burgoine, Lorraine Mumtaz, Mike Rayner

The Data Availability Statement appearing with the published article is incorrect. The correct Data Availability Statement should read:

There are legal restrictions on the student outcome data. National Child Measurement Programme data was used as determined by the data sharing agreement with the then Information Centre of the National Health Service (NHS IC), now Health and Social Care Information Centre (HSCIC). The data sharing agreement stated that all record level data must be treated in accordance with the Data Protection Act 1998 and individual level data must not be made available without agreement of the NHS IC. Access to data from the National Child Measurement Programme was granted to Nick Townsend during his previous employment with the National Obesity Observatory (now part of Public Health England). All analysis of the data was undertaken then and the data was not seen by any other authors of this paper. Sharing of the NCMP data is governed by a set of regulations (http://www.legislation.gov.uk/uksi/2013/218/pdfs/uksi_20130218_en.pdf) which state that outside of Department of Health and Public Health England, data can only be disclosed in a form in which no individual child can be identified. Cleaned and anonymised NCMP data has subsequently been made available by the HSCIC through the UK Data Service. Please see below for the contact information of this third party. The food outlet data from the local councils of West Berkshire are provided in supplemental files. All food outlet data are attributed to their respective local councils: Bracknell Forest Council (http://www.bracknell-forest.gov.uk), Reading Borough Council (http://beta.reading.gov.uk/home), Slough Borough Council (https://www.slough.gov.uk/), The Royal Borough of Windsor and Maidenhead (http://www.rbwm.gov.uk/contacts.htm), Wokingham Borough Council (http://www.wokingham.gov.uk/), West Berkshire Council. To apply for individual-level NCMP data, please contact the Health and Social Care Information Centre,enquiries@hscic.gov.uk.
